# Who Differentiates by Skin Color? Status Attributions and Skin Pigmentation in Chile

**DOI:** 10.3389/fpsyg.2019.01516

**Published:** 2019-07-03

**Authors:** Fernanda Torres, Mauricio Salgado, Bernardo Mackenna, Javier Núñez

**Affiliations:** ^1^Institute of Sociology, Pontificia Universidad Católica de Chile, Santiago, Chile; ^2^School of Social Sciences, Faculty of Education and Social Sciences, Universidad Andres Bello, Santiago, Chile; ^3^Department of Sociology, University of California, San Diego, San Diego, CA, United States; ^4^Department of Economics, Faculty of Economics and Business, Universidad de Chile, Santiago, Chile

**Keywords:** Chile, inequality, skin pigmentation, status beliefs, status construction theory, whiteness

## Abstract

A growing body of research has shown that phenotypes and skin pigmentation play a fundamental role in stratification dynamics in Latin American countries. However, the relevance of skin color on status attribution for different status groups has been little studied in the region. This article seeks to broaden the research on phenotypic status cues using Chile as a context for analysis – a Latin American country with a narrow although continuous spectrum of skin tones, marked status differences, and a mostly white elite. We draw on status construction theory to hypothesize that skin pigmentation in Chile has become a status cue, although its heuristic relevance could differ across status groups. Using visual stimuli and a repeated measure design, we studied this relationship and tested whether the use of skin pigmentation as a status cue is conditional upon the status of those categorizing others. The results reveal that participants attribute, on average, lower status to others of darker skin. Besides, skin pigmentation has a conditional effect on the social status of participants: whereas skin pigmentation does not work as a status cue for lower status participants, it is an important status marker for the categorizations that middle and especially higher status participants perform. The phenotypic composition of reference groups of low- and high-status individuals and system justification are discussed as potential explanations for these results.

## Introduction

In interactions with strangers, individuals resort to available information that their culture and experiences indicate are relevant for assessing the person they are interacting with (Goffman, 1959). Appearance, and in particular facial appearance, is often marked in ways that reveal membership of different social categories, such as gender, race, or social class (Fişek et al., 2005). These markers, or cues, help people form *first impressions* (Willis and Todorov, 2006): the process of categorizing others and inferring attributes relevant to the interaction. Status cues are indicators associated with actors that provide information to others about their social status. They can be classified according to two orthogonal dimensions: (1) indicative-expressive and (2) task-category (Fişek et al., 2005). The first dimension is concerned with *how* status information is communicated: indicative cues explicitly identify or label a person as possessing some status (e.g., a diploma); expressive cues are implicit, so they provide indirect status information (e.g., skin pigmentation). The second dimension considers *what* status information is communicated: task cues inform “what these people can do”; categorical cues communicate “who these people are” (ethnicity, race, or class). But the importance of specific status cues for social categorization depends not only on who possesses them; it also depends on the heuristic relevance they have for those who perform the categorization (Berger et al., 1972). This means that social categorization may also *vary interpersonally*: certain visual markers can be relevant for some, but not for others – i.e., a status cue can be conditional upon the observers’ characteristics. Empirical research has established that individuals categorize the status of others with great precision using visual cues (Mazur, 1993; Pape et al., 2012). In this article, we address the categorization process in the status hierarchy that individuals perform using facial appearance, as occurs in everyday life. Specifically, our objective is to understand the importance that skin pigmentation has for categorization on the status ladder, in a country with a continuous (although narrow) spectrum of skin pigmentation, profound status differences, and a mostly white elite, like Chile.

Status is a fundamental part of the inequality levels present in any society (Ridgeway, 2014). In Chile, inequality has been a structuring feature of the social order throughout history (PNUD, 2017). Chile is one of the most unequal countries in the world, with a Gini index of 46.6 points (The World Bank, 2017). There is currently an enormous concentration of wealth in the highest levels of Chilean social hierarchy (López et al., 2013), a social mobility regime characterized by high elite closure (Torche, 2005), low intergenerational mobility (Núñez and Miranda, 2010), and clear occupational barriers between social classes (Espinoza and Núñez, 2014).

Evidence suggests that a powerful status cue in Chile is ancestry, marked by surnames (Núñez and Gutiérrez, 2004). However, additional and more obvious attributes for categorizing the status of others in daily life have been poorly studied. It is remarkable that, for instance, the significance of racial traits such as skin pigmentation in social categorization has not been thoroughly studied in the local social sciences. Although this is a research bias for most Latin American countries (Telles and Flores, 2013), in Chile, this epistemological obstacle for racial research is largely explained by the historical presence of a *race-blind* ideology (Walsh, 2015), which has permeated the social sciences in the country (Barandiarán, 2012). According to this ideology, intensive miscegenation, which has occurred since colonial times, dissolved most of the racial differences, bringing about “one of the most uniform races of the entire world” (Walsh, 2015). Only recently, empirical research has shown the importance of race in general, and skin pigmentation in particular, for status attribution in Chile (Lizcano, 2005; Staab and Maher, 2006; Salgado and Castillo, 2018). All in all, this race-blind ideology has also permeated the culture, so although the Chilean population is genetically diverse (Eyheramendy et al., 2015), the majority self-identify as white, and they manifest strong preferences for light-skinned Chileans, even those who self-identify as *morenos* (Uhlmann et al., 2002).

Pronounced inequality, a narrow but continuous spectrum of skin pigmentation (CIIR, 2017) and limited knowledge on the subject, make Chile an interesting case study for social categorization attributes. Most studies have been conducted in developed societies with marked racial differences (e.g., Mazur, 1993; Pape et al., 2012). Also, current research has usually focused on analyzing status cues, without inquiring into whether their use and relevance varies interpersonally. This article aims to fill this gap in the literature using Chile as the study context. To do so, we used a set of photographs of post-secondary students (i.e., students attending universities, vocational training centers, and technical school centers) from different social backgrounds, which were later categorized by three independent samples of raters (all of them also post-secondary students). Then, through a repeated-measures design and a sampling procedure that maximized the heterogeneity of the status of participants, we requested an independent sample of post-secondary Chilean students to categorize the social status of the photographees.

## Status Categorizations Based on Visual Information

The most obvious carrier of status markers is the body; hence, it is fundamental for status attributions. The body is shaped by the cultural context (Le Breton, 2002), so ascribed markers such as skin pigmentation or acquired ones such as clothing styles implicitly communicate “who the person is” (i.e., they are expressive-categorical social cues). According to Goffman (1959), when individuals present themselves, others resort to visual information to facilitate interaction. In encounters with strangers, individuals pay attention to markers that indicate socio-economic status (SES), competence, or moral integrity. Individuals know this, so they build a personal *façade* with elements that can be intimately related to themselves. Studies have shown that individuals are able to “see” – based solely on visual information – the status affiliation of others (Mazur, 1993; Pape et al., 2012). Facial appearance is particularly relevant in this process. Different studies have shown that a person’s face is used to infer various characteristics, such as their physical attractiveness, amiability, competence, trustworthiness, and aggressiveness (Oosterhof and Todorov, 2008; Rivera, 2010; Eckel and Petrie, 2011).

### Raters’ Social Status

Status attribution, as well as being context specific, is *interpersonally variable*. The identities of the observers, or *raters*, influence their perception, and therefore, the social status they attribute to others (Rivera, 2010). According to Bourdieu (1984), class-specific *habitus* not only leaves its mark on the configuration of an individual’s lifestyle and appearance, but also influences his/her perception and classification of the lifestyle and appearance of others. In this way, the habitus organizes the practices and the perception of practices. The disposition of individuals, structured by the habitus, depends on their positions within the social hierarchy. Since Merton (1968), social scientists have known that social categorization depends on the reference group (i.e., family, friends, co-workers). Merton understood the reference group as “a context for evaluating the relative position of oneself and others” (Merton, 1968, 338). Thus, the reference group provides a structure for comparison by which individuals value themselves and others.

Although self-positioning in the status hierarchy (a subjective measure of status) tends to be biased toward the scale mean, it also depends on factors related to the individual’s objective status, such as income, occupation, or schooling (Evans and Kelley, 2004; Castillo et al., 2013). High-status individuals tend to underestimate their own status, while low-status individuals tend to overestimate theirs, so on average the population places itself near the middle of the social hierarchy. This “centripetal” force toward the mean value is brought about because self-positioning reflects the *subjective sampling* of individuals from their own reference group. Due to structural constraints or personal preferences, most people interact and cluster with people who are similar to themselves in significant ways (McPherson et al., 2001). Thus, reference groups tend to be fairly homogenous with respect to social status. This status homogeneity means that most individuals are objectively near the average of their reference group. This objective centrality, in turn, encourages people to see themselves as middle-status. According to Evans and Kelley (2004), subjective sampling is a special case of the “availability heuristics” (Tversky and Kahneman, 1973), a systematic perceptual bias whereby individuals base their perceptions on their immediate social milieu. Social contexts in which the elite is highly segregated from the rest of the population, as in the Chilean case, can strengthen this perceptual bias (Castillo et al., 2013).

Since the status categorization of others could also be biased considering its relational nature, it is necessary to empirically analyze whether personal status (either self-reported or objective) has an effect on status attribution. It is likely that as personal status increases, individuals attribute, on average, a higher status to others – and vice versa – because their status evaluations appear to be framed or anchored in their own status position (i.e., anchored in their own reference group). This led us to formulate the first research hypothesis:

H1: The higher the social status of the rater, the greater the social status attributed to others.

### Skin Pigmentation

Skin color is also a status cue. Skin pigmentation may act as an expressive-categorical status cue: it can implicitly communicate who the person is. In Latin America, Telles (2014) observed that skin pigmentation is a fundamental stratification variable; a source of social discrimination and inequality. People in Latin America usually attribute a higher social status to those who have lighter skin tones, an association that has developed historically in the continent. White pigmentation is thus an ascribed, inalienable symbolic capital, conceived as a legacy which gave high status groups the right to superiority (Telles and Flores, 2013; Telles and Paschel, 2014). The importance of whiteness in the continent is such that skin color conservation mechanisms have been put into practice, as in the marriage market, illustrating the preference for marrying people with white skin pigmentation (Collier and Sater, 2004; Telles and Flores, 2013). In Chile, white people also tend to enjoy formal and informal privileges, social deference, and attribution of socially valued properties (Uhlmann et al., 2002; Salgado and Castillo, 2018). Therefore, it is likely that dark-skinned individuals in Chile are categorized as lower status compared to light-skinned ones. Thus, our second hypothesis states that:

H2: The darker the skin pigmentation of others, the lower the social status attributed to them.

However, the importance of skin pigmentation for social categorization could also vary interpersonally: some people might use skin pigmentation as an expressive-categorical status cue, but others might not (Berger et al., 1972). Many factors can explain this variability, but there are structural and cultural reasons for predicting that the heuristic value of skin pigmentation can be conditional upon the social status of raters. As Telles (2014) pointed out, racial stratification in Latin America is far from that of the United States. Instead of well-defined racial clusters – straightforwardly associated with social status – present in the latter, the former has gone through an extensive miscegenation process that has created a racial gradient with subtle differences, except for the higher echelons of society, which tend to be white. In Chile, previous research suggests that status evaluations of wealth are highly correlated with whiteness (Salgado and Castillo, 2018). Also, studies of skin pigmentation in the metropolitan region (CIIR, 2017) have shown that skin color appears stratified in the Chilean capital: individuals from wealthier households tend to be whiter and have less diversity in skin color than other status groups. These skin-pigmentation differences in group composition could cause the heuristic value of this racial attribute for status categorization to differ for higher and lower status individuals. Thus, for instance, we expect higher status participants to perceive lighter skinned individuals as “ingroup members” (and, therefore, of higher status), and darker skinned individuals as “outgroup members” (therefore, lower status), due to subjective sampling (Kahneman and Tversky, 1972) from their more homogenous reference group. That is, skin pigmentation should be an available heuristic for higher status individuals in status attributions. It is more difficult to predict whether skin pigmentation has a heuristic value for lower status participants in status categorization, given the higher heterogeneity of this racial trait within their reference group. Since high-status individuals tend to be white in Chile, while lower status groups are more heterogeneous (although, on average, darker), we predict that skin color might be a relevant status cue only for high-status groups. Thus, we can expect that:

H3: The heuristic value of skin pigmentation is conditional upon the status of the individual performing the categorization.

In this study, we categorized the visual stimuli (i.e., portraits of young people) using a continuous measure of skin pigmentation rather than ethnoracial categories. This methodological decision rests on previous research in Latin America and Chile, which has shown that ethnoracial categories are inappropriate for explaining stratification regimes, mobility dynamics, and the stereotyping process in person perception. This is mainly due to the within-category racial or color heterogeneity present in Latin American ethnic groups. Telles et al. (2015) demonstrated that skin pigmentation measures are better at capturing the racial inequalities of these countries. Hence, Telles (2014) has dubbed the Latin American status hierarchies as “pigmentocracies.” Besides, research on stereotyping has shown that “racial ambiguity” (specific racial traits combined with different skin pigmentations) leaves more room for judgmental biases in person perception (Caruso et al., 2009). Skin color could therefore also better explain the social categorization of others in the Latin American context. In recent research on stereotyping conducted in Chile, participants (undergraduate students) estimated the wealth of adolescent faces present in visual stimuli (Salgado and Castillo, 2018). In that study, researchers categorized the stimuli in ethnoracial groups (e.g., Amerindian, White, Black, *Mulato*). It was shown that participants tended to stereotype Amerindians (as poor) and Whites (as wealthy), but the status attributions were more diffuse in the other ethnoracial groups. Thus, ethnoracial categories did not adequately explain all the variance in the data. However, one continuous dimension provided a better explanation of data variability in status attributions: a “whiteness index,” an *ad hoc* measure of the percentage of raters that classified each picture in the stimuli as White. Hence, in this study, we decided to use a more continuous, fluid, and regionally validated measure of skin pigmentation, namely: the PERLA skin color palette (Telles, 2013), designed to evaluate skin color specifically in Latin America. It is made of eleven skin tones: the number 1 corresponds to the lightest complexion and 11 to the darkest complexion.

### Confounding Factors

There are additional factors that can affect status attribution to others. In this research, we controlled for several confounding variables that the literature identifies as important in the status categorization of others. *Physical attractiveness* is also related to status categorization. Faces considered attractive are associated with the upper strata of society (Webster and Driskell, 1983; Mazur, 1993). Moreover, people considered physically attractive are assumed to excel in “objective” qualities unrelated to appearance (Langlois et al., 2000; Zebrowitz et al., 2002; Hosoda et al., 2003). *Perceived competence* is another relevant dimension for status categorization (Fiske et al., 2007). Pape et al. (2012) showed that status attribution depends to a great extent on the levels of intelligence (or competence) perceived by raters: those perceived as intelligent are attributed a higher social status. Besides, the documented stereotype that individuals in less prestigious roles do not have the level of intelligence of individuals in successful roles serves as an ideological justification for socio-economic differences (Fiske et al., 2002), thus rationalizing and justifying social inequality (Jost, 2001). Status construction theory also suggests that *compensatory characteristics* influence status beliefs: low-status groups are usually associated with less valued but positive qualities, such as *generosity* and *trustworthiness* (Ridgeway et al., 1998; Ridgeway and Correll, 2006). This is also in line with the formation of stereotypes, which combine warmth and competence according to social status (Fiske et al., 2002, 2007). Finally, visible accessories (such as glasses, necklaces, earrings, and baseball caps) and the clothing that individuals wear in daily life might also serve as a signal of status group membership (Mattan et al., 2017; Oh et al., 2018). In this study, we controlled for the effects of all these variables among photographees.

## Materials and Methods

**FIGURE 1:**
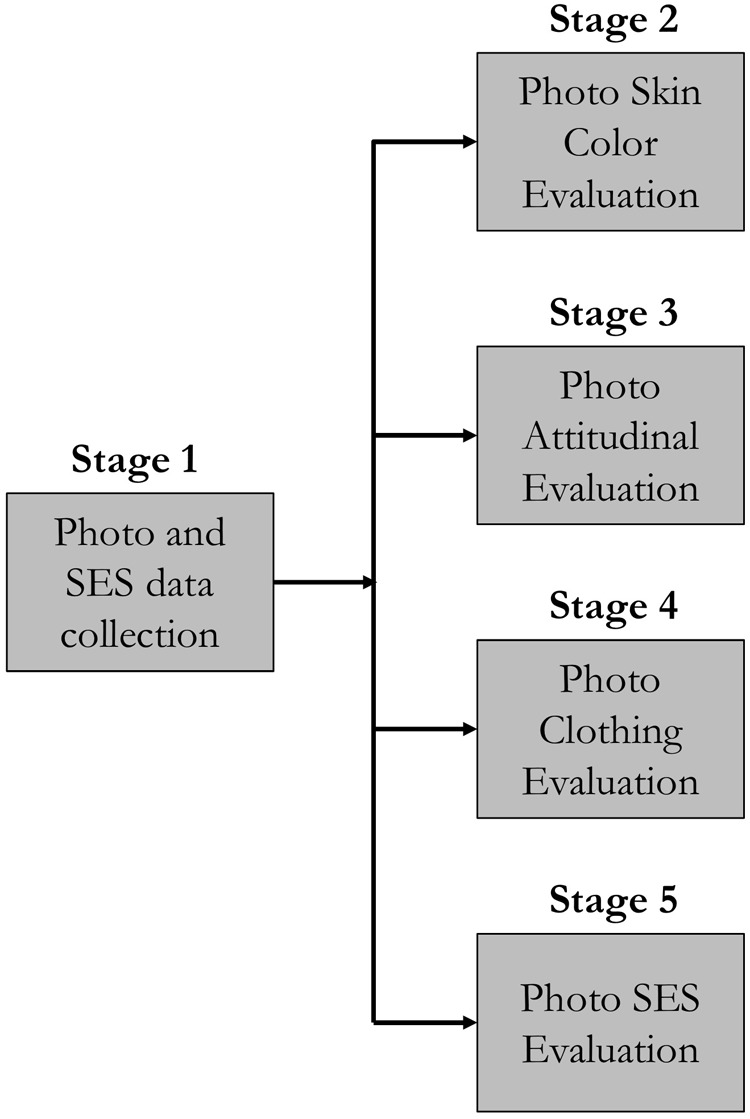
Data collection stages.

Data were collected in four stages, which are depicted in Figure 1. A total of 120 photographs of higher education students were obtained in Stage 1. The students voluntarily accepted their photographs being used in this study by signing an informed consent. The photographs were taken at four post-secondary institutions in Santiago (Metropolitan Region), Chile: (i) a popular vocational training center, (ii) a technical school center, (iii) an unselective university, and (iv) a highly selective university. Considering the strong socio-economic stratification of the Chilean education system, these institutions were selected with the aim of maximizing the social heterogeneity among the photographees. Photographs were taken as participants were dressed that day, in order to portray them as they present themselves to others in everyday life. For this reason, photographs with facial alterations such as hats, glasses, make-up, or jewelry were not excluded, considering that both phenotypic and intentional characteristics form the personal *façade* (Goffman, 1959).^1^ Following photograph selection protocols from previous research (e.g., Rule and Ambady, 2008), the only requirements for compiling the set of photographs were (i) a balanced sample had to be obtained in terms of gender (50% of those photographed were women), (ii) the facial expression of those photographed had to be neutral, and (iii) the face had to be looking directly at the camera. In addition, photographees answered a socio-economic characterization survey; thus, self-reported information was available to characterize the sample in terms of gender, age and social status. The average age of the 120 students was 21 (SD = 2.42).

In Stage 2, 32 students from two different universities in Santiago (a private and a public university) evaluated the 120 photographs using the PERLA color palette (Telles, 2013). Students were invited to participate in a “classification of photographs” study in the computer laboratories at the two universities. Volunteers were introduced and trained in the PERLA color palette, and then evaluated the skin pigmentation of the photographees’. Hence, there were a total of 3,840 evaluations for the 120 photographs. The average age of the 32 participants was 20.6 (SD = 2.48) and 56% were female. In Stage 3 of data collection, a survey was sent via e-mail to students from a university with campuses in the three most populated Chilean regions (Valparaíso, Concepción, and the Metropolitan Region) to evaluate ten randomly selected photographs from the 120 pictures. A total of 2,104 raters took part in this stage, so there were 180 evaluations per photograph, approximately. Evaluations consisted in attributing a certain level of physical attractiveness, competence, generosity, and trustworthiness to the photographee. The average age of this sample of raters was 23.8 (SD = 6.55) and 62% of participants were female. In Stage 4,^2^ we requested a sample of 32 students – attending (i) a highly selective university and (ii) a technical training center – to categorize the status of the clothing of the 120 photographees on the MacArthur scale of subjective social status (Adler et al., 2000). In this stage, the photographs were altered to hide phenotypical traits (such as hair and facial features) and leave only visible accessories (e.g., necklaces, earrings, baseball caps) and clothing. This evaluation enabled us to gauge the status attributed to the visible clothing in the picture. There were a total of 3,840 assessments of the 120 photographs. The average age of the 32 participants was 21 (SD = 3.24) and 57.58% were female. A sample of the altered photographs used in this *ex post* assessment can be found in Supplementary Figure S2A.

In Stage 5, an independent sample of 271 higher education students, belonging to (i) a vocational training center and (ii) a highly selective university, categorized the status of the photographees. In this stage, the study followed a repeated measures design, in which each rater categorized the status of 30 random photographs on the MacArthur scale (Adler et al., 2000). Thus, each photograph was rated 70 times approximately, with 8,130 ratings in total. In this stage, participants were paid pocket money for turning up. Also, to elicit “true” social categorization (i.e., status beliefs), we incentivized response-precision by giving a gift-card valued at $30,000 pesos (around US$ 47) to the top three raters whose assessments best coincided with the actual status of the photographees (based on the socio-economic characterization survey of Stage 1). Hence, raters had a financial stake in the status they were predicting. To reduce carry over effects typical of repeated measures designs, participants rated stimuli presented at random. Raters also answered a socio-demographic questionnaire. The average age of the 271 participants was 21 (SD = 2.65), similar to the photographed subjects; 36.9% of raters were female. The self-reported mean in the MacArthur subjective status scale was 5.48 (SD = 1.66).

The central argument in this study is that status attribution is not only determined by the characteristics of the raters (i.e., their own status) but also by cultural markers present in the facial features of the photographees (i.e., their skin pigmentation), and by the interaction between these two variables (i.e., the conditional effect on status attribution of skin pigmentation and the status of raters). Besides, being a repeated measures design, it may be possible that the status attributions of raters are related – responses come from the same participants regarding the same stimuli – which could bias standard errors in linear OLS models. Hence, in order to analyze the status attributions raters assigned to the photographs, we modeled the data using linear mixed-effect models – which account for the patterns of non-independence present in the data (Judd et al., 2012). In the regression models, level 1 corresponds to the status categorizations of raters – the ratings of the 271 participants in Stage 4. Level 2 corresponds to the (attributed) characteristics of photographs, evaluated in Stages 2 and 3 of data collection (we used the average ratings for each photograph). Hence, status attributions are nested in the photographs, which are treated as random, and there are 8,130 cases at level 1 (categorizations or ratings) and 120 cases at level 2 (photographees’ attributes). Level 2 variables included the skin pigmentation of the photographees, (average) rating, and all the covariates, namely: physical attractiveness, competence, generosity, and trustworthiness. All these variables were treated as fixed characteristics of the photographs.

### Dependent Variable: Status Attributions

We adapted the MacArthur scale of subjective social status (Adler et al., 2000) to categorize the status of others. Thus, each rater categorized the photographee, responding on a scale of 1 to 10, to the following question: “In terms of social status, there are groups of people in Chile that tend to be located at the highest levels of society, while others are located at the lowest levels. On a scale of 1 (lowest level) to 10 (highest level), where would you place the photographed subject?” Table 1 shows the main descriptive results of this variable.

**Table 1:** Descriptive statistics for dependent and independent variables.

Statistics	*N*	Average	SD	Min	Max	Source
**Dependent variables**						
Attributed status	8,130	5.6	1.67	1	10	Stage 5
**Level 1 independent variables**						
Subjective status of raters	8,130	5.48	1.67	1	10	Stage 5
Objective status of raters	7,560	1.93	0.90	0	3.65	Stage 5
Age of raters	8,130	21.09	2.65	18	36	Stage 5
**Level 2 independent variables**						
Perceived skin pigmentation of photographees	120	2.83	1.02	1.16	5.59	Stage 2
Perceived physical attractiveness of photographees	120	4.14	0.90	2.45	6.74	Stage 3
Attributed competence of photographees	120	6.20	0.57	4.65	7.40	Stage 3
Attributed generosity of photographees	120	5.73	0.65	3.98	7.15	Stage 3
Attributed trustworthiness of photographees	120	5.55	0.66	3.87	6.99	Stage 3
Attributed status of clothing and accessories	120	5.24	0.79	3.41	7.13	Stage 4

### Independent Variables at Level 1: Subjective and Objective Status of the Raters

Literature distinguishes between objective and subjective SES measures. While objective SES corresponds to economic and social position in relation to others (usually measured by income, education, and occupation indicators), subjective SES is the individual’s conception of a person’s position compared to the positions of others. Since the issue of which SES measure is more relevant for studying subjective experiences is contentious in the literature (Kraus and Stephens, 2012), in this study, we have included both measures as independent variables. Thus, on the one hand, as a subjective status measure, individual raters had to position themselves on the social status ladder using the MacArthur scale, responding on a gradation of 1 to 10. On the other hand, as an objective measure of status, we carried out a principal component factorial analysis to construct the objective SES variable of raters, which comprises the father’s occupation, mother’s occupation (both occupations codded according to the *International Standard Classification of Occupations, ISCO-08*, see International Labour Organization, 2012), father’s education, and mother’s education.^3^ Thus, we will be able to test the effects of an individual’s objective and subjective SES on status attributions to others. Table 1 shows the main descriptive results of this variable.

### Independent Variables at Level 2: Skin Pigmentation

The PERLA color palette (Telles, 2013) was used to measure the skin pigmentation of our sample of photographs^4^ during Stage 3 of data collection. We used the average skin pigmentation score for each photograph. Table 1 shows the main descriptive results.

### Control Variables

In Stage 2, a sample of evaluators judged perceived physical attractiveness, competence (i.e., intelligence), generosity, and trustworthiness on a scale ranging from 1 (none whatsoever) to 10 (considerable) for each attribute. We also included dummy variables that indicate the presence or absence in the picture of some accessories, such as glasses necklaces, earrings, and baseball caps. Finally, because the pictures left the upper torso and some clothing visible, we added a measure of attributed status based only on the visible clothing (altering the photographs by covering skin and hair color, see footnote 2 in this paper). Averages were used in the evaluations of each photographee based on these appraisals. Table 1 shows the main descriptive results of these variables.

## Results

We fitted four mixed linear models, which randomize intercepts to allow variation between photographs in the participants’ social status attributions.^5^ To analyze the more relevant characteristics in status attribution, standardized coefficients were also estimated, which make the variables comparable in their effect. Model results are shown in Table 2, including two additional covariates of raters (gender and age) and one additional covariate of photographees (gender). Table 2 also displays three goodness-of-fit statistics: the *log-likelihood*, the *Akaike’s information criterion* (AIC), and the *Bayesian information criterion* (BIC) statistics. Lower AIC and BIC values across nested models indicate better fit. Finally, Table 2 also shows, as adjustment measures, the coefficient of determination for generalized linear models proposed by Nakagawa et al. (2017): the marginal and conditional *R*^2^ values. The mixed models were fitted using the *lme4* statistical package (Bates et al., 2015) in R. The *p*-values for the fixed effects were estimated using Satterthwaite’s method.

**Table 2:** Mixed regression models.

	Model 1			Model 2			Model 3			Model 4		
Level 1: Raters	Coef.	EE	*Z*	Coef.	EE	*Z*	Coef.	EE	*Z*	Coef.	EE	*Z*
Subjective status	0.17***	(0.01)	0.17				0.28***	(0.04)	0.28			
Objective status				0.05**	(0.02)	0.03				0.27***	(0.07)	0.14
Age	–0.04***	(0.01)	–0.06	–0.04***	(0.01)	–0.06	–0.04***	(0.01)	–0.06	–0.04***	(0.01)	–0.06
Gender (ref: male)	–0.32***	(0.03)	–0.09	–0.30***	(0.03)	–0.09	–0.31***	(0.03)	–0.09	–0.30***	(0.03)	–0.09
**Level 2: Photographees**												
Skin pigmentation	–0.15**	(0.05)	–0.09	–0.15**	(0.05)	–0.09	0.06	(0.07)	0.04	0.02	(0.05)	–0.01
Physical attractiveness	0.51***	(0.08)	0.27	0.50***	(0.08)	0.27	0.49***	(0.07)	0.26	0.46***	(0.07)	0.25
Competence	0.16	(0.18)	0.05	0.16	(0.18)	0.05	0.14	(0.17)	0.05	0.11	(0.17)	0.04
Generosity	–0.77***	(0.22)	–0.30	–0.77***	(0.23)	–0.30	–0.70***	(0.21)	–0.27	–0.68**	(0.21)	–0.26
Trustworthiness	0.70*	(0.28)	0.27	0.71*	(0.28)	0.28	0.64*	(0.26)	0.25	0.64*	(0.26)	0.25
Gender (ref: male)	–0.64***	(0.12)	–0.19	–0.64***	(0.13)	–0.19	–0.62***	(0.12)	–0.19	–0.58***	(0.11)	–0.17
Glasses (ref: does not have)	–0.11	(0.14)	–0.02	–0.11	(0.14)	–0.02	–0.08	(0.13)	–0.01	–0.08	(0.13)	–0.02
Necklace (ref: does not have)	0.03	(0.10)	0.00	0.03	(0.11)	0.00	0.07	(0.10)	0.01	0.12	(0.10)	0.03
Earring (ref: does not have)	–0.24*	(0.10)	–0.06	–0.22*	(0.11)	–0.05	–0.25*	(0.10)	–0.07	–0.20*	(0.10)	–0.05
Cap (ref: does not have)	–0.25	(0.18)	–0.05	–0.24	(0.18)	–0.05	–0.26	(0.17)	–0.05	–0.19	(0.17)	–0.04
Status attributed to visible clothing	0.28***	(0.07)	0.13	0.30***	(0.07)	0.14	0.26***	(0.07)	0.12	0.26***	(0.06)	0.12
*Interaction effects*												
Subjective status × skin pigmentation							–0.04**	(0.01)	–0.18			
Objective status × skin pigmentation										–0.08**	(0.02)	–0.15
**Variance components**												
Level 2 variance (intercept)	0.19			0.19			0.22			0.10		
Residual variance	1.96			2.03			1.93			2.00		
Marginal *R*^2^/conditional *R*^2^	0.22/0.31			0.20/0.28			0.19/0.29			0.16/0.26		
Log likelihood	–14,409.90			–13,532.66			–14,382.06			–13,503.60		
AIC	28,853.79			27,099.31			28,804.11			27,047.21		
BIC	28,972.85			27,217.13			28,944.18			27,185.82		
Number of photographees	120			120			120			120		
Number of raters’ responses	8,130			7,560			8,130			7,560		
Number of raters	271			252			271			252		

*Dependent variable: social status attributed to the photographee. ^∗∗∗^*p* < 0.001, ^∗∗^*p* < 0.01, ^∗^*p* < 0.05. Standard errors are in parentheses. *Z* statistics correspond to standardized regression coefficients.*

Model 1 in Table 2 shows that the higher the subjective status of raters, the greater the status attributed to the photographee (b = 0.17, p < 0.001), controlling for the other variables in the model. Model 2 was adjusted with the same variables but using the objective (instead of subjective) status of raters as predictors. This model provides a robustness test for the findings. The relationship of the objective status of raters is equivalent to that found for the subjective status in Model 1: the greater the objective status of raters, the higher the status attributed to the pictured subject (b = 0.05, p = 0.007). These results support our first hypothesis, which predicted that the higher the status of the rater, the higher the status assigned to others, over and above other confounding factors at the levels of raters and photographees. Hence, the results suggest that participants categorize the status of others from their own relative position in the socioeconomic ladder: they seem to compare and construct the status of others using their own reference groups, so their status attributions are anchored in their own status position. Besides, the positive relationship between the status of raters and status attributions to others is present whether the subjective or objective status of raters is considered. Nevertheless, the regression coefficient in Model 1 indicates that subjective status is a stronger predictor of status attributions among raters, compared to objective status in Model 2.

Our second hypothesis stated that the darker the skin pigmentation of photographees, the lower the social status attributed to them. To test this hypothesis, Model 1 and Model 2 also include, as a fixed effect of the photographees, their average skin pigmentation. Both models show that the skin pigmentation of photographees is negatively related to the status attributions of raters, controlling for the other variables in the model (Model 1: b = -0.15, p = 0.002; Model 2: b = -0.15, p = 0.002). These results support our research Hypothesis 2. Hence, skin pigmentation in Chile is an important factor in the process of categorizing the status of others, confirming that darker skin tones are associated with lower status positions, as recent evidence in Latin America and in Chile has shown (Uhlmann et al., 2002; Telles, 2014; Salgado and Castillo, 2018).

**FIGURE 2:**
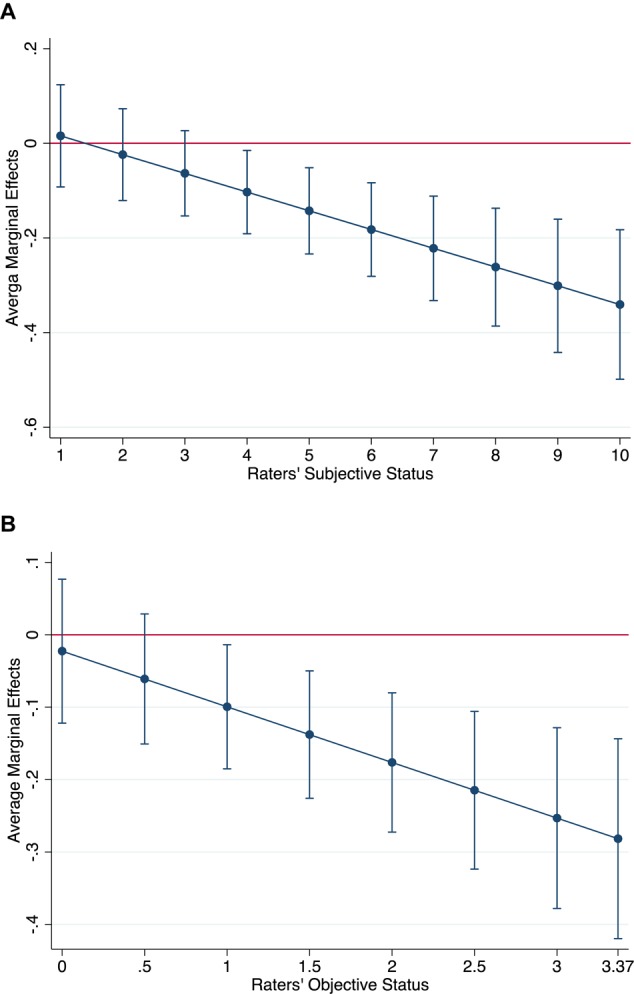
Average marginal effects of the skin pigmentation of photographees on status categorization according to subjective **(A)** and objective status **(B)** of the rater. Bars indicate 95% confidence intervals.

The question is, however, whether the heuristic value of skin pigmentation is conditional upon the status of the individual performing the categorization, as stated by our research Hypothesis 3. Surveyed research in Chile including skin color (LAPOP, 2016; CIIR, 2017) indicates that higher status individuals tend to have lighter skin pigmentation. Thus, skin pigmentation could act as a meaningful status cue for the wealthiest raters. We tested this conditional effect in Model 3 and Model 4 as an interaction (i.e., multiplicative) effect: while Model 3 includes the statistical interaction between the subjective status of raters and the skin pigmentation of photographees, Model 4 evaluates the same interaction effect, but using the objective status of raters.^6^ We thus evaluated whether the status categorizations are interpersonally variable; that is, whether they depend on the attributor’s identity (Rivera, 2010). Table 2 indicates that these interaction effects, both in Model 3 (b = -0.04, p = 0.002) and Model 4 (b = -0.08, p = 0.002), are statistically significant. Besides, the difference between Model 3 and Model 1 in AIC is ΔAIC = -49.7 and in BIC is ΔBIC = -28.67, while the difference between Model 4 and Model 2 in AIC is ΔAIC = -52.1 and in BIC is ΔBIC = -31.31, which suggest that the inclusion of the interaction effects improves the model’s fit. Besides, when considering the interaction effects between the subjective and objective status of the rater with the skin pigmentation of the photographee in Model 3 and Model 4, respectively, the direct effect of skin pigmentation diminishes (compared to Model 1 and Model 2), becomes imprecisely estimated, and thus insignificant at conventional levels (Model 3: b = 0.06, p = 0.40; Model 4: b = 0.02, p = 0.69). These results suggest that, as Hypothesis 3 states, the effect of the skin pigmentation of the photographees on status attributions is conditional upon the status of the rater. To further analyze these results, we post-estimated the average marginal effects in Stata 15 using the command *margins*. Figure 2 shows the results of this analysis.

Figure 2A shows the average marginal effect of skin pigmentation on attributed status, according to the subjective status of raters. First, it can be seen that the effects of skin pigmentation on status attribution are statistically insignificant when the status of the raters is low. Statistically significant interaction effects appear for raters who self-positioned greater than or equal to 4 on the social status ladder. As the status of the raters increases, the effects of skin pigmentation on social status intensify – i.e., skin pigmentation becomes more relevant to status attribution. Additionally, the marginal effect diminishes as the subjective status increases, which indicates that a darker complexion in the photographs leads to lower status categorization for those raters with higher subjective status. Figure 2B shows the interaction effects between the objective SES of raters and the skin pigmentation of photographees on status attribution. The conclusion is the same: regardless of whether we analyze subjective or objective SES, skin pigmentation becomes negatively related to status attributions as the status of raters increases. Thus, it seems that skin pigmentation has been constituted in Chile as an element of distinction for mid- and high-status groups: skin tone functions as a status cue only for them.

The estimated relationships (see Table 2) of the control variables at the stimuli level are also worth mentioning. In line with previous research, the physical attractiveness of photographees was positively related (and significantly at conventional levels) to the status attributions of raters across models. Previous research on status attribution has found that people considered physically attractive are assumed to excel in qualities unrelated to appearance (Langlois et al., 2000; Hosoda et al., 2003). Our results are inconclusive with regard to competence. In our four models, the coefficients were small, imprecisely estimated, and thus insignificant at conventional levels. Finally, regarding the compensatory characteristics in status beliefs (Ridgeway and Erickson, 2000), the photographs categorized as low-status were those with greater perceived generosity across models. This is also in line with previous research indicating that those with less resources tend to give more (Piff et al., 2010). However, faces perceived as more trustworthy were attributed *higher* social status, across all models, a result that contradicts extant evidence regarding compensatory characteristics. Previous research has shown that trustworthiness is related to the dimension of *warmth*, which is generally attributed to people with lower social status as opposed to characteristics related to competence, which, as mentioned above, are associated with higher status individuals (Fiske et al., 2007). Interestingly, compensatory characteristics (i.e., generosity and trustworthiness) had the greatest influence in either diminishing or increasing the social status attributions of photographees across models, as assessed by the standardized regression coefficients. The gender of photographees also had an influence on the status attributions of raters across models: being female led to lower status categorization than being male. This result is also in line with previous research suggesting that gender is a *diffuse status cue* (Auspurg et al., 2017): gender creates (and activates) cultural beliefs in the higher competence and status worthiness of men, thus entitling them to higher rewards.

We ran an additional robustness check to assess the stability of the reported results regarding the moderating effect of skin pigmentation on the relationship between the status of raters and their status attributions to the pictures. Since raters came from two different educational institutions (a strategy we employed to maximize the status heterogeneity in the sample), the role of attendance of these institutions on status attributions may not be clear. To address this potential concern, we adjusted additional regression models by mingling the raters’ educational institutions and their status attributions (see Supplementary Table S2A). The post-estimation analysis of a three-way interaction effect revealed that there was no major difference in the reported results between raters attending the highly-selective university and the vocational training center (see Supplementary Figure S4A).

## Discussion

In this paper, we have studied the status attributions individuals assign in Chile, a country with a continuous (although narrow) spectrum of skin pigmentation, profound status differences, and a mostly white elite. We have focused our analysis on two factors which, our results suggest, are interwoven in status categorization: the (subjective and objective) status of individuals, and the skin pigmentation of others. Using visual stimuli (120 photographs of Chilean post-secondary students) and a repeated measure design, we tested three hypotheses: whether higher status individuals assign higher status to others; whether skin pigmentation acts as a status cue; and whether the heuristic value of the skin pigmentation of others in status categorization is conditional upon the social position of raters in the status hierarchy. Our results could be summarized as follows: first, we found a positive relationship between the status of individuals and the status they attribute to others. Thus, the process of status attributions seems to be anchored in personal status: individuals evaluate others from their own reference group, so higher status individuals tend to attribute higher status to others, while lower status individuals assign, on average, lower status to others. Second, the results show a negative relationship between darker skin pigmentation of others and the status attributed to them, i.e., skin pigmentation works as a status cue to categorize others in the social hierarchy. Third, skin pigmentation does interact with the status position of those categorizing others, over and above other confounding factors that are relevant in status categorization. That is, the heuristic value of skin pigmentation as a status cue is conditional upon the status of those categorizing others. Our results show that, whereas skin pigmentation does not work as a status cue for lower status individuals, it is an important status marker for the categorization performed by mid- and high-status individuals. Indeed, in our study, higher status individuals tended to attribute lower status to those with darker skin, and higher status to those with lighter skin.

The finding that skin pigmentation is mainly used by middle and (more markedly) high-status individuals to categorize the status of others has profound implications. Some potential and complementary explanatory mechanisms are evaluated below.

First, among the Chilean elite, whiteness could be a distinction marker because this is a phenotypic trait present in most of their peers. There are historical and sociological reasons for the formation of a light-skinned elite in Chile. For instance, although this country experienced more than three centuries of racial miscegenation, members of the elite self-excluded themselves from this process during the 19th century. For over a century, the Chilean “aristocracy” had an implicit rule: they were open to marriage with non-elites who had not participated in *mestizaje*; those who, even if poor, had just arrived from Europe (Collier and Sater, 2004). Thus, in choosing a marriage partner, whiteness mattered more than status to Chilean elites. Nowadays, Chile is quite exceptional in the Latin American context. According to the America’s Barometer data, Chile is both the whitest and least diverse among the 26 countries measured (LAPOP, 2016). And the same survey data also show that Chile has the whitest and least diverse elites in the sub-continent – tied in homogeneity with the much darker skinned Nicaragua. Also, studies of skin pigmentation in Santiago, the Chilean capital (CIIR, 2017), have shown that skin color appears highly stratified: individuals from richer households tend to be whiter and have smaller variance in skin pigmentation than other status groups. The fact that Chile has this particularly white and uniform elite might explain our findings that skin color *has become* a powerful status cue for higher status individuals, especially for the elites: because their peers are mostly white, they distinguish themselves from the rest by this phenotypic marker.

Second, since the reference groups of non-elite individuals are more varied in terms of skin tone, this attribute could not work as an available status cue for them. Again, historical and sociological reasons might help to explain the conformation of this racial heterogeneity. For instance, since colonial times, there has been certain social mobility for blacks, mulattos, and indigenous people, who have achieved middle-class positions – through employment in the army during the 18th and 19th century (Contreras, 2011) or through the school system during the 20th century (Salgado and Castillo, 2018) – sharing the same social space with light-skinned or white-looking Chileans. The higher phenotypic variance in middle and lower status groups might provide the subjective experience that weakens the link between skin pigmentation and social status.

Third, it is also possible that lower status individuals do not use skin pigmentation as a status cue because they believe this ascribed trait should not affect the social position of a person in a meritocratic society. Consistent with *system justification theory* (Jost, 2001), neglecting the value of skin pigmentation as a status cue may help young Chileans in post-secondary institutions to defend the ideological integrity of the social system in which they live, even at the expense of personal and group interests. Previous research has shown that young people from indigenous and low-income backgrounds in Latin America are more likely to endorse meritocratic beliefs (Henry and Saul, 2006) than their white and privileged peers. In this way, low-status students may reduce the cognitive dissonance of living in a country in which, the dominant ideology claims, the social status of any person depends only on their effort and decisions – an ideology that stresses the achieved dimension of status (Araujo and Martuccelli, 2014) – while experiencing the symbolic advantage of ascribed, inherited traits that only the Chilean elites possess, such as their ancestry and, as the results presented here indicate, their lighter skin pigmentation. On the other hand, there would be no corresponding ambivalence among high-status individuals, because their status position and group composition (mostly white peers) can seamlessly coexist with a meritocratic ideology.

For the low-status participants in this study – who do suffer the consequences of an unequal country in which whiteness is an ascribed symbolic capital – not using skin pigmentation as a status cue might be an expression of their endorsement of the meritocratic ideology that has dominated the public sphere in Chile in the last 40 years; a way of reestablishing a sense of confidence in the promise of upward mobility. Indeed, national surveys show high conformity with current living conditions in Chile, and endorsement of the meritocratic discourse among young people (between 15 and 29 years of age): most Chilean youth are highly satisfied with their lives (88%), and most of them believe that dispositional factors (e.g., hard work) are more important than contextual factors (e.g., having good contacts) for success in life (INJUV, 2015). Based on these data, some authors have concluded that Chilean youth culture is characterized by a *conformist majority* – who are culturally integrated to the Chilean variety of capitalism (Brunner, 2017).

All in all, our results are silent to these potential explanations. However, we believe these explanations (group reference composition and system justification theory) provide indications for further research in this area. What precise mechanism explains the reported statistical interaction between the social status of raters and the skin pigmentation of photographees in status categorizations remains obscure, because the analyzed data are observational in nature. Additional experimental and cross-cultural research (for instance, using implicit rather than explicit predictors of status attribution) is needed to test the proposed explanatory mechanisms, or alternative ones. Besides, some caveats deserve mention. First, the main measures in this study were observed, not manipulated, so the main findings are correlational rather than causal. Second, portraits were taken in natural conditions, so they included elements such as hairstyles, accessories, and clothing, which could confound our findings. Nonetheless, this strategy allowed us to study status attributions to more naturalistic targets, taking into account the way in which targets present themselves in everyday life (Goffman, 1959). Also, in the regression models, we included as control variables the presence of accessories (baseball caps, earrings, and neckless) and the status attributed to the targets’ clothing by an independent sample, hence the status attributions of raters are adjusted for those elements. Therefore, given the implemented research design, the chosen analytical strategy, and the cultural characteristics of the context in which we carried out our research, we are confident that the main results presented here are robust. The findings offered here are a starting point for additional research on skin pigmentation and status beliefs in the region.

## Ethics Statement

This study was carried out in accordance with the recommendations of the National Commission for Scientific and Technological Research (CONICYT) of Chile, with written informed consent from all subjects. All subjects gave written informed consent in accordance with the Declaration of Helsinki. The protocol was approved by the Research Ethics Committee at the Universidad Andrés Bello.

## Author Contributions

MS, JN, BM, and FT conceived and designed the analysis and collected the data. FT and BM performed the analysis. FT, MS, and BM wrote the manuscript.

## Conflict of Interest Statement

The authors declare that the research was conducted in the absence of any commercial or financial relationships that could be construed as a potential conflict of interest.

## Footnotes

1 A sample of photographs used in the study is available on the journal web page as Supplementary Material.

2 We carried out this *ex post* evaluation following one of the reviewer’s concern regarding possible confounds (which provided visual information about targets beyond skin tone, such as accessories and clothing).

3 In the questionnaire, parents’ education had the alternative “I do not know,” which was excluded from the analyses. Therefore, the total number of ratings (i.e., attributions) in the regression models that include raters’ objective status is different from the models that include the raters’ subjective status. Details on the exploratory factor analysis are available on the journal web page as Supplementary Material (see Supplementary Table S1A).

4 It is worth noting that our photo sample has similar skin tone values to the LAPOP (2016) national representative sample, which includes a measurement on the same scale. In fact, the mean skin tone in our sample is statistically undistinguishable even from subsets of the LAPOP sample, such as younger age cohorts, or northern region, where one would expect darker skin tones, by virtue of it being closer to the equator. Supplementary Figure S3A provides such comparisons.

5 Furthermore, in terms of intra-class correlation, it seems pertinent to use this type of model, since an intra-class correlation coefficient (ICC) of 0.216 was obtained.

6 We also fitted a linear mixed model in which both subjective and objective status were included as predictors, and the multiplicative term was estimated using photographees’ skin pigmentation and raters’ subjective status. The results are similar to those reported in Table 2.
