# Supplementary material for: Who Differentiates by Skin Color? Status Attributions and Skin Pigmentation in Chile
Source: Front Psychol. 2019 Jul 3;10:1516. doi: 10.3389/fpsyg.2019.01516 (PMC6618139; doi:10.3389/fpsyg.2019.01516)
Supplement: Supplementary file 1 [file Table_1.DOCX]

**Supplemental Materials**


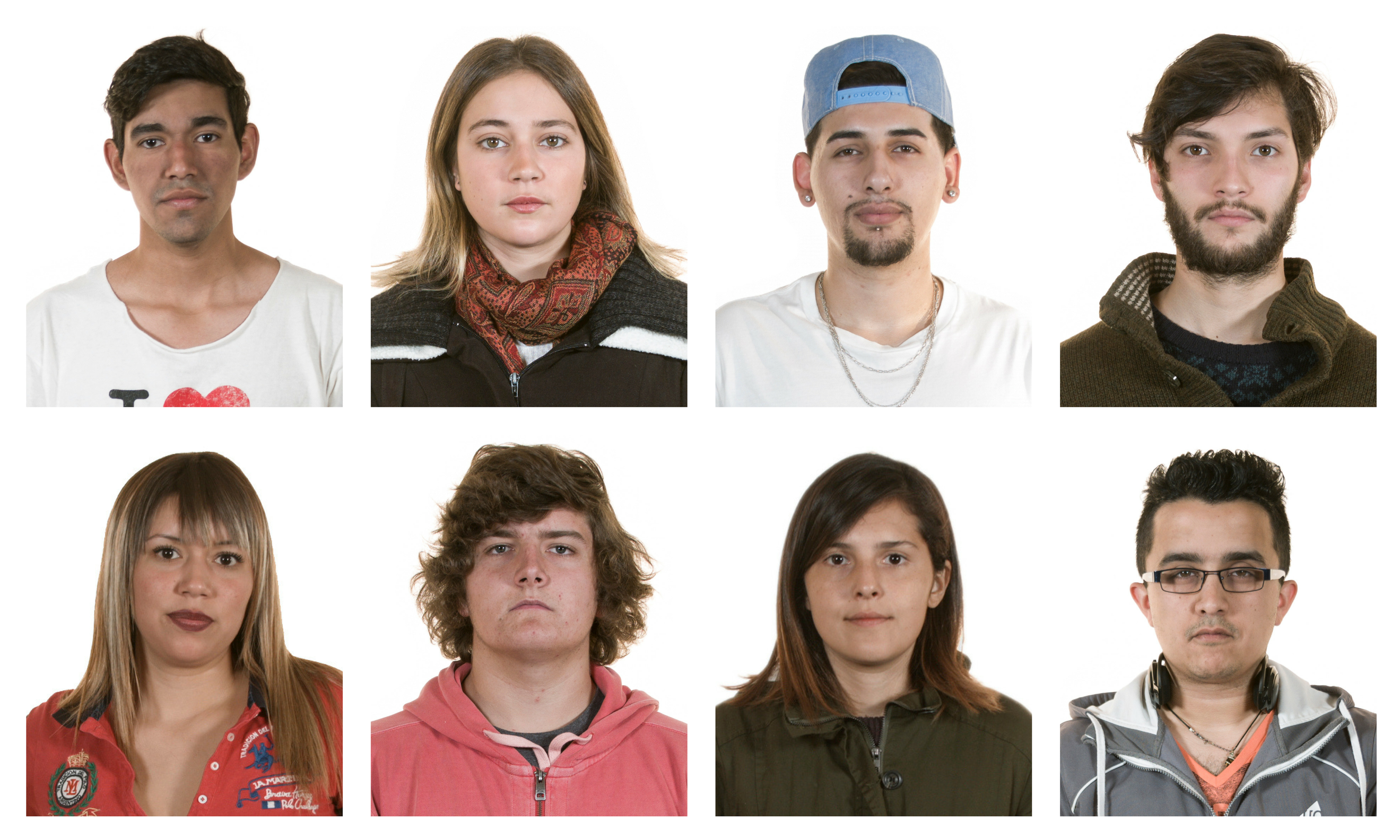


**Figure 1A**: Sample of the photographs used in the study (i.e., Stages 2, 3, and 5)


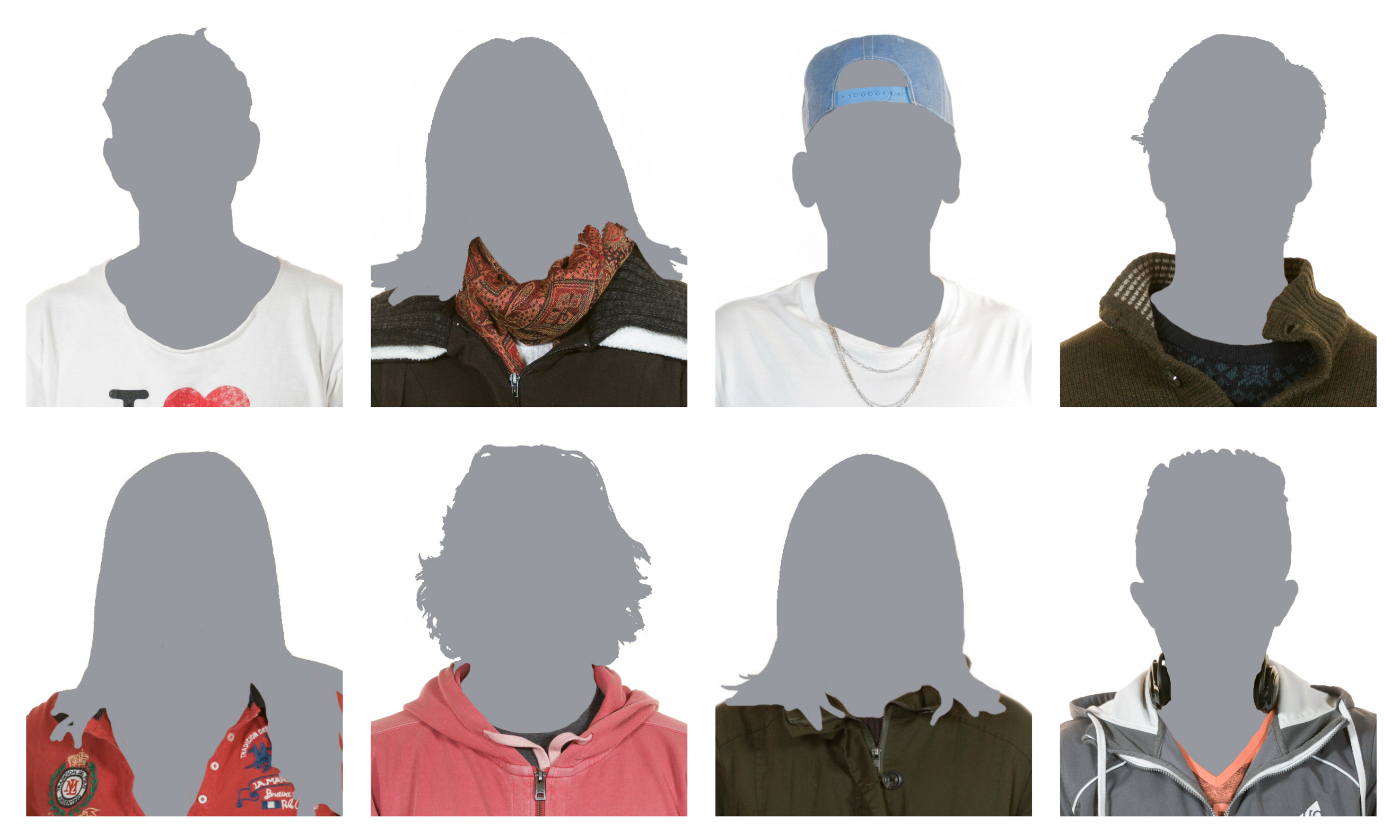


**Figure 2A**: Sample of the altered photographs used in Stage 4 of data collection

| **Table 1A**. Construction of raters’ objective status:  Factor loadings and unique variances | | | |
| --- | --- | --- | --- |
| Variable | Factor 1 | Factor 2 | Uniqueness |
| Father’s Occupation | 0.6968 | -0.1862 | 0.4797 |
| Mather’s Occupation | 0.6288 | 0.2490 | 0.5426 |
| Father’s Education | 0.7643 | -0.1859 | 0.3813 |
| Mother’s Education | 0.7934 | 0.1453 | 0.3494 |
| Kaiser-Meyer-Olkin measure of sampling adequacy | | | |
| Variable | KMO | | |
| Father’s Occupation | 0.7645 | | |
| Mather’s Occupation | 0.7295 | | |
| Father’s Education | 0.7075 | | |
| Mother’s Education | 0.7121 | | |
| Overall | 0.7260 | | |

**Figure 3A**: Means of skin color in our sample of Photos, compared to estimates from the nationally representative LAPOP 2016 survey, with 95% CI intervals, considering design effects.


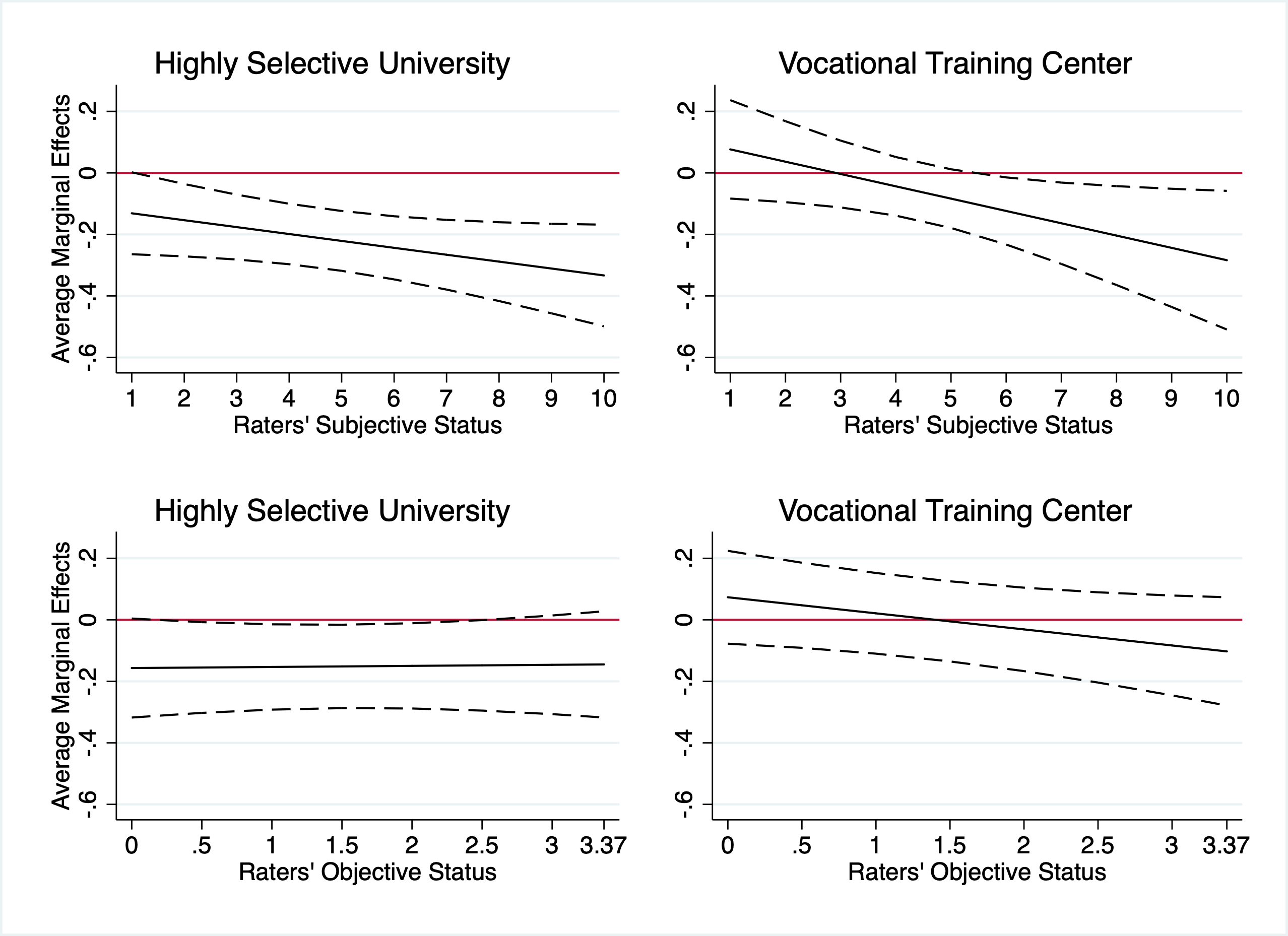


**Figure 4A**: Average marginal effects of the skin pigmentation of photographees on status categorization according to subjective (upper panel) and objective status (lower panel) of the raters attending the Highly Selective University and the Technical School Centre. Dashed lines indicate 95% confidence intervals.

| **Table 2A**. **Mixed regression models. Dependent variable: Social status attributed to the photographee** | | | | | | | | | | | |
| --- | --- | --- | --- | --- | --- | --- | --- | --- | --- | --- | --- |
| Model / Predictor | 1 | 2 | 3 | 4 | 5 |  | 6 | 7 | 8 | 9 | 10 |
| Level 1: Raters | Coef.  (SE) | Coef.  (SE) | Coef.  (SE) | Coef.  (SE) | Coef.  (SE) |  | Coef.  (SE) | Coef.  (SE) | Coef.  (SE) | Coef.  (SE) | Coef.  (SE) |
| Subjective status | 0.207*** | 0.319*** | 0.207*** | 0.285*** | 0.269*** |  |  |  |  |  |  |
|  | (0.012) | (0.033) | (0.011) | (0.034) | (0.038) |  |  |  |  |  |  |
| Objective status |  |  |  |  |  |  | 0.140*** | 0.358*** | 0.140*** | 0.260*** | 0.075 |
|  |  |  |  |  |  |  | (0.027) | (0.073) | (0.026) | (0.078) | (0.088) |
| Age | -0.025*** | -0.025*** | -0.025*** | -0.025*** | -0.025*** |  | -0.029*** | -0.030*** | -0.030*** | -0.030*** | -0.031*** |
|  | (0.006) | (0.006) | (0.006) | (0.006) | (0.006) |  | (0.006) | (0.006) | (0.006) | (0.006) | (0.006) |
| Gender (ref. male) | -0.240*** | -0.239*** | -0.240*** | -0.240*** | -0.240*** |  | -0.248*** | -0.247*** | -0.248*** | -0.248*** | -0.215*** |
|  | (0.032) | (0.032) | (0.032) | (0.032) | (0.032) |  | (0.034) | (0.034) | (0.034) | (0.034) | (0.034) |
| Vocational Training Center (ref. University) | 0.455*** | 0.454*** | 0.023 | 0.077 | -0.226 |  | 0.353*** | 0.353*** | -0.080 | -0.017 | 0.008 |
|  | (0.033) | (0.033) | (0.093) | (0.095) | (0.347) |  | (0.038) | (0.038) | (0.103) | (0.110) | (0.248) |
| Level 2: Photographees |  |  |  |  |  |  |  |  |  |  |  |
| Skin Pigmentation | -0.097* | 0.052 | -0.196*** | -0.079 | -0.109 |  | -0.104* | -0.024 | -0.213*** | -0.153* | -0.157 |
|  | (0.045) | (0.061) | (0.049) | (0.069) | (0.077) |  | (0.044) | (0.051) | (0.050) | (0.062) | (0.082) |
| Physical attractiveness | 0.485*** | 0.484*** | 0.486*** | 0.485*** | 0.484*** |  | 0.456*** | 0.457*** | 0.458*** | 0.458*** | 0.451*** |
|  | (0.074) | (0.073) | (0.073) | (0.073) | (0.073) |  | (0.072) | (0.072) | (0.072) | (0.072) | (0.103) |
| Competence | 0.125 | 0.126 | 0.128 | 0.128 | 0.128 |  | 0.096 | 0.101 | 0.101 | 0.102 | 0.128 |
|  | (0.170) | (0.169) | (0.169) | (0.169) | (0.169) |  | (0.166) | (0.166) | (0.165) | (0.165) | (0.240) |
| Generosity | -0.651** | -0.667** | -0.654** | -0.663** | -0.666** |  | -0.667** | -0.676** | -0.669** | -0.672** | -0.500 |
|  | (0.212) | (0.212) | (0.211) | (0.211) | (0.212) |  | (0.208) | (0.208) | (0.207) | (0.207) | (0.298) |
| Trustworthiness | 0.582* | 0.600* | 0.585* | 0.596* | 0.599* |  | 0.623* | 0.632* | 0.623* | 0.627* | 0.422 |
|  | (0.267) | (0.266) | (0.265) | (0.265) | (0.266) |  | (0.260) | (0.260) | (0.259) | (0.260) | (0.374) |
| Gender (ref. male) | -0.591*** | -0.595*** | -0.593*** | -0.595*** | -0.594*** |  | -0.564*** | -0.567*** | -0.567*** | -0.567*** | -0.533** |
|  | (0.117) | (0.117) | (0.117) | (0.117) | (0.117) |  | (0.114) | (0.114) | (0.114) | (0.114) | (0.165) |
| Glasses | -0.061 | -0.063 | -0.062 | -0.063 | -0.063 |  | -0.067 | -0.071 | -0.069 | -0.070 | -0.002 |
|  | (0.132) | (0.131) | (0.131) | (0.131) | (0.131) |  | (0.129) | (0.129) | (0.129) | (0.129) | (0.185) |
| Necklace | 0.070 | 0.069 | 0.071 | 0.072 | 0.072 |  | 0.126 | 0.122 | 0.125 | 0.124 | 0.115 |
|  | (0.099) | (0.099) | (0.099) | (0.099) | (0.099) |  | (0.097) | (0.097) | (0.097) | (0.097) | (0.139) |
| Earring | -0.260** | -0.259** | -0.259** | -0.259** | -0.259** |  | -0.199* | -0.199* | -0.199* | -0.199* | -0.256 |
|  | (0.099) | (0.099) | (0.099) | (0.099) | (0.099) |  | (0.097) | (0.097) | (0.097) | (0.097) | (0.139) |
| Cap | -0.261 | -0.259 | -0.257 | -0.256 | -0.256 |  | -0.186 | -0.187 | -0.185 | -0.185 | -0.224 |
|  | (0.174) | (0.173) | (0.173) | (0.173) | (0.173) |  | (0.170) | (0.170) | (0.170) | (0.170) | (0.244) |
| Status attributed to visible clothing | 0.246*** | 0.248*** | 0.248*** | 0.249*** | 0.249*** |  | 0.243*** | 0.246*** | 0.246*** | 0.246*** | 0.211* |
|  | (0.066) | (0.066) | (0.066) | (0.066) | (0.066) |  | (0.065) | (0.065) | (0.065) | (0.065) | (0.093) |
| Interaction Effects |  |  |  |  |  |  |  |  |  |  |  |
| Subjective status * Skin pigmentation |  | -0.039*** |  | -0.027* | -0.022 |  |  |  |  |  |  |
|  |  | (0.011) |  | (0.011) | (0.013) |  |  |  |  |  |  |
| Objective status * Skin pigmentation |  |  |  |  |  |  |  | -0.077** |  | -0.042 | 0.004 |
|  |  |  |  |  |  |  |  | (0.024) |  | (0.026) | (0.029) |
| VTC * Skin pigmentation |  |  | 0.152*** | 0.133*** | 0.225 |  |  |  | 0.153*** | 0.131*** | 0.230** |
|  |  |  | (0.031) | (0.032) | (0.116) |  |  |  | (0.034) | (0.036) | (0.083) |
| VTC * Subjective status |  |  |  |  | 0.058 |  |  |  |  |  |  |
|  |  |  |  |  | (0.064) |  |  |  |  |  |  |
| VTC * Subjective status * Skin pigmentation |  |  |  |  | -0.018 |  |  |  |  |  |  |
|  |  |  |  |  | (0.021) |  |  |  |  |  |  |
| VTC * Objective status |  |  |  |  |  |  |  |  |  |  | 0.019 |
|  |  |  |  |  |  |  |  |  |  |  | (0.122) |
| VTC * Objective status * Skin pigmentation |  |  |  |  |  |  |  |  |  |  | -0.056 |
|  |  |  |  |  |  |  |  |  |  |  | (0.040) |
| Level 2 Variance (Intercept) | 0.255*** | 0.264*** | 0.260*** | 0.263*** | 0.264*** |  | 0.290*** | 0.297*** | 0.294*** | 0.295*** | 0.343*** |
|  | (0.054) | (0.052) | (0.053) | (0.052) | (0.052) |  | (0.045) | (0.044) | (0.044) | (0.044) | (0.078) |
| Residual Variance | 1.963** | 1.518* | 2.218*** | 1.878** | 1.968** |  | 3.145*** | 2.880*** | 3.415*** | 3.238*** | 2.833** |
|  | (0.667) | (0.676) | (0.666) | (0.680) | (0.688) |  | (0.653) | (0.658) | (0.655) | (0.664) | (0.941) |
| Marginal R2 / Conditional R2 | 0.25/0.72 | 0.25/.072 | 0.25/0.72 | 0.25/0.72 | 0.25/0.72 |  | 0.22/0.72 | 0.22/0.72 | 0.22/0.72 | 0.22/0.72 | 0.23/0.72 |
| Log Likelihood | -14297 | -14294 | -14288 | -14288 | -14294 |  | -13464 | -13462 | -13457 | -13458 | -13382 |
| AIC | 28632 | 28629 | 28615 | 28618 | 28633 |  | 26967 | 26964 | 26953 | 26958 | 26809 |
| BIC | 28765 | 28769 | 28755 | 28765 | 28794 |  | 27098 | 27103 | 27091 | 27104 | 26968 |
| Number of Raters’ Responses | 8,130 | 8,130 | 8,130 | 8,130 | 8,130 |  | 7,560 | 7,560 | 7,560 | 7,560 | 7,560 |
| Number of Photographees | 120 | 120 | 120 | 120 | 120 |  | 120 | 120 | 120 | 120 | 120 |
| Number of Raters | 271 | 271 | 271 | 271 | 271 |  | 252 | 252 | 252 | 252 | 252 |
| Unstandarized coeficients reported. Standard errors in parentheses. *** p<0.001, ** p<0.01, * p<0.05 | | | | | | | | | | | |
